# Editorial: Cross-boundary significance of methanogens - the methane moment and beyond

**DOI:** 10.3389/fmicb.2024.1434586

**Published:** 2024-06-24

**Authors:** Zhe Lyu, Amelia-Elena Rotaru, Mark Pimentel, Cui-Jing Zhang, Simon K.-M. R. Rittmann, James G. Ferry

**Affiliations:** ^1^Department of Plant and Microbial Biology, North Carolina State University, Raleigh, NC, United States; ^2^Nordic Center for Earth Evolution (NORDCEE), University of Southern Denmark, Odense, Denmark; ^3^Medically Associated Science and Technology (MAST) Program, Cedars-Sinai, Los Angeles, CA, United States; ^4^Archaeal Biology Center, Institute for Advanced Study, Shenzhen University, Shenzhen, China; ^5^Archaea Physiology & Biotechnology Group, Department of Functional and Evolutionary Ecology, Universität Wien, Wien, Austria; ^6^Arkeon GmbH, Tulln a.d. Donau, Austria; ^7^Department of Biochemistry and Molecular Biology, Pennsylvania State University, University Park, PA, United States

**Keywords:** methanogenesis, microbiome, host, environment, agriculture, energy, health, biotechnology

## Introduction

As previously outlined in our editorial preface (Lyu et al.), methanogens are anaerobic methane-producing archaea that colonize almost every corner of the earth. This ubiquitous distribution is coupled with their enormous diversity, outsized contributions to global methane emissions, active interactions with surrounding microbiomes, and potential roles in the health and disease of humans, animals, and plants. Consequently, it is our collective view that methanogens are of cross-boundary significance, a primary motivation for us to establish this exciting Research Topic.

In conclusion, we are very fortunate to have been able to edit and publish 18 manuscripts from 122 authors. Here, our editorial team presents an epilog for a bird's-eye view of these manuscripts. Because this Research Topic is dedicated to William “Barny” Whitman, our epilog ends with a special commentary about him, contributed by James G. Ferry.

Emeritus Professors Whitman and Ferry have both advanced methanogen research in their own remarkable ways, and we invite scientists around the world to stand on their and other giants' shoulders to continue pushing boundaries in studying this powerful group of archaea. Together, we shall create a collective momentum that helps to carry humanity through the methane moment and beyond.

## Summary of the Research Topic

Studies from this Research Topic reinforce that methanogens occupy diverse niches across the globe. Broadly, these methanogens can be classified as environmental or host-associated. Using culture-independent and/or culture-based approaches, our authors have found environmental methanogens, both established and new species, in habitats often deemed challenging for many other life forms. These include a saline and alkaline soda lake in Russia (Khomyakova et al.), sulfate-rich marine sediments in the USA (Coon et al.), heavily polluted landfill sites in India (Prakash et al.), an alkaline terrestrial mud volcano in Russia (Khomyakova et al.), coal beds at moderate (400 m) and high (800–1,200 m) depths in China (Fu et al.) and India (Chawla et al.), and deep (640–1,738 m) underground gas storage facilities in Czech Republic and Slovakia (Hanišáková et al.). Collectively, these findings not only help us better understand the contributions of environmental methanogens to global methane emission and their adaptation strategies in diverse habitats, but offer potential applications of methanogens in solid waste treatment (Prakash et al.), lithium biorecovery from saline waters (Azim et al.), biogas recovery from coal beds (Fu et al.; Chawla et al.), and Power-to-Gas technology (Hanišáková et al.).

Compared to their environmental counterparts, host-associated methanogens are very much understudied, but our authors are beginning to narrow this knowledge gap. First, two comprehensive reviews on rumen (Khairunisa et al.) and mammalian (Volmer et al.) methanogens summarize the current knowledge, highlight the limitations therein, and offer a roadmap to address these limitations. Second, using multi-omics approaches, two experimental studies on rumen (Malik et al.) and porcine (Meene et al.) microbiomes provide rare functional insight of cattle-, buffalo-, and swine-associated methanogens in the context of diet treatment and viral infection. Finally, a molecular survey of the microbiomes across 11 multicellular species reveals positive methanogen signatures in 4 species including humans, nematode, and two sponge species (von Hoyningen-Huene et al.). The nematode discovery is of high significance, suggesting that the model organism *Caenorhabditis elegans* may be adopted to study host-methanogen interactions for the health and diseases of humans and animals.

Regardless of where the methanogens are found, they must eventually be studied by employing laboratory models for a mechanistic understanding of their biology and potential applications. Our authors are also making advances here across multiple scales. At the DNA level, a user-friendly CRISPR/Cas12a toolbox for genome editing is developed for *Methanosarcina acetivorans*, targeting regions beyond what the popular Cas9 reaches, especially the T-rich sites (Zhu et al.). Advancing to the protein level, the function of a methylthiotransferase shared by both methanogens and methanotrophic archaea is determined to catalyze the methylthiolation of select tRNAs (Boswinkle et al.). In closed batch cultures, a unified basal medium is created for methanogens. When supplemented with differential substrates and gases, it enables growth of three methanogen species operating different methanogenesis pathways (Ngoumelah et al.). Expanding to bioreactors, sophisticated cultivation methods are optimized to achieve fast, high density, and large-scale growth of *Methanococcus maripaludis* in fed-batch cultivation mode (Palabikyan et al.), bringing it one step closer to feasible biotechnology applications for sustainable biomanufacturing.

Last but not least, we must pursue unified principles that govern the biology of methanogens. Equally important is to update existing principles as necessary. Our authors set excellent examples on both fronts. Emeritus Professor Conrad, another giant in our field, theorizes that regardless of the ecological niches and the microbiome composition therein, the rate limiting microbes for ecosystem methane production are often methanogens instead of the primary fermenters, because methanogens exhibit unusually high apparent activation energy (*E*_*a*_). Given that the higher the *E*_*a*_ the faster a metabolism becomes when temperatures rise, this theory predicts that methanogenesis will increase more rapidly than most other metabolisms as the atmosphere warms, thus contributing more to global warming. In another example, Professor Mukhopadhyay reminds us of a common misconception that “pseudomerein” or “pseudopeptidoglycan” instead of peptidoglycan occur in archaea, specifically the *Methanobacteria* and *Methanopyri*. The short answer is that these “pseudo” cell wall polymers are indeed a specific type of peptidoglycan, which should simply be named archaeal peptidoglycan in contrast to the bacterial types. This shall renew the interest of developing archaeal-specific antibiotics for human and animal use, as existing peptidoglycan-targeted drugs are only effective on bacteria.

## Dedication to Prof. Whitman by Prof. Ferry

It is particularly fitting that this volume is dedicated to the career of William “Barny” Whitman who has made exceptional and lasting contributions to the field of methanogenesis both in research and service. Barny's research has produced milestone advances in physiological and ecological understanding achieved by integrating genetics, molecular biology, and biochemistry. His early discovery of nickel in cofactor F_430_ of methylcoenzyme M reductase (Whitman and Wolfe, 1980; Ellefson et al., 1982), and subsequent biochemical characterization of the enzyme (Whitman and Wolfe, 1983, 1987), launched a path taken by him and other investigators leading to current understanding of this key enzyme essential to all methanogenic pathways (Lyu et al., 2018, 2020; Shao et al., 2022). His biochemical contributions extend much further including anabolic and catabolic understanding of *Methanococci*. Equally significant, he developed genetic approaches for *Methanoccoci* that greatly accelerated a broad understanding and initiated a broader application of genetics in methanogen research. Barny's willingness to share expertise and collaborate has furthered an understanding of *Methanococci* that cannot be overstated. Collectively, Barny along with his trainees and collaborators have contributed ~120 publications in archaeal research focusing on methanogens.

Barny is also recognized for his major contributions to the broader field of microbiology including both the domain *Bacteria* and *Archaea*. Of note is his highly cited article entitled “Prokaryotes: The unseen majority” (Whitman et al., 1998) that raised awareness of microbiology to the broader scientific community, his integrated approach researching the marine roseobacteria and soil bacteria (Singleton et al., 2001; Furlong et al., 2002; Upchurch et al., 2008; Reisch et al., 2011; Bullock et al., 2017; Wirth et al., 2020), and his leadership in the newly established SeqCode (Hedlund et al., 2022) as a nomenclatural code for uncultivated prokaryotes.

Barny has given tirelessly to service benefiting methanogen research and the broader microbial field. For the past three decades, he has been the main driving force clarifying the taxonomy of methanogens (Keswani et al., 1996; Liu and Whitman, 2008; Prakash et al., 2023; Cui et al., 2024). It would be remiss to not recognize his wider contributions as supervising editor of Bergey's Manual and editor of Archaea. With the exceptional width and depth, it is challenging to summarize Professor Whitman's accomplishments in a single sentence except to recognize that the strength of the methanogen field would be glaringly less without his contributions. Indeed, the same applies to the wider field of prokaryotic biology.

## Author contributions

ZL: Writing – review & editing, Writing – original draft. A-ER: Writing – review & editing. MP: Writing – review & editing. C-JZ: Writing – review & editing. SK-MRR: Writing – review & editing. JF: Writing – review & editing, Writing – original draft.
